# A case report of *de novo* ELANE c.416C>T mutation causing cyclic neutropenia in a 7-year-old girl

**DOI:** 10.3389/fimmu.2026.1815284

**Published:** 2026-04-14

**Authors:** Rongjie Chen, Zhe Xu, Fu Zhang

**Affiliations:** 1 Department of Pediatrics, Guangyuan Central Hospital, Guangyuan, Sichuan, China; 2 Department of Pediatrics, Guangyuan Central Hospital Affiliated to North Sichuan Medical College, Nanchong, Sichuan, China

**Keywords:** cyclic neutropenia, *de novo* mutation, diagnosis and treatment, ELANE gene, pediatric

## Abstract

**Objective:**

Childhood cyclic neutropenia (CN) is a rare hematopoietic disorder. We describe the clinical phenotype, diagnostic features, and management of a pediatric CN case caused by a *de novo* ELANE mutation, to improve clinical recognition and care.

**Methods:**

We retrospectively analyzed clinical, laboratory, and genetic data from a 7-year-old girl with CN, combined with bioinformatics and literature review to explore genotype–phenotype correlations and optimal therapy.

**Results:**

The patient presented with classic 21−day cyclic episodes of fever, oral ulcers, and lymphadenopathy. Peripheral blood absolute neutrophil count (ANC) fell to 0.04×10^9^/L at nadir, accompanied by monocytosis. Whole−exome sequencing identified a *de novo* heterozygous ELANE c.416C>T (p.Pro139Leu) variant. The Pro139 residue is highly conserved across species, and substitution is predicted to impair neutrophil elastase structure and function. The patient responded well to recombinant human granulocyte colony−stimulating factor (rhG−CSF) plus infection control and supportive care and is currently awaiting hematopoietic stem cell transplantation (HSCT).

**Conclusion:**

Pediatric CN features periodic fever and mucosal infection. Diagnosis requires both cyclic neutropenia documented by serial monitoring and pathogenic ELANE variant confirmation. rhG−CSF remains first−line therapy. Identification of this *de novo* c.416C>T variant broadens the genotypic spectrum of ELANE-related CN. Early and accurate diagnosis, individualized treatment, and long−term follow−up are critical to improve outcomes.

## 1 Introduction

Cyclic neutropenia (CN) and severe congenital neutropenia (SCN) are major autosomal dominant primary immunodeficiencies caused by *ELANE* mutations (1–4). The hallmark of CN is periodic fluctuation of peripheral blood ANC, typically with a 21−day cycle (range 14–35 days). At nadir, ANC usually drops below 0.2×10^9^/L and recovers spontaneously within 3–6 days (5–7). Although *ELANE* mutations underlie both CN and SCN, the molecular mechanism driving periodic ANC oscillations in CN remains unclear (5, 8).

CN is an extremely rare hematologic disorder, with an incidence of 1–6 per million (9, 10). Most patients develop symptoms in infancy or childhood, with rare adult−onset cases (7, 11–13). Its low prevalence and nonspecific early presentation often lead to misdiagnosis or diagnostic delay (14). Many children undergo unnecessary invasive tests (e.g., repeated bone marrow aspiration) for persistent neutropenia, causing physical distress and substantial family burden (15–18).

To strengthen clinical awareness and diagnostic capacity for pediatric CN, we retrospectively analyzed a 7−year−old patient with CN, integrating clinical, laboratory, and genetic findings with bioinformatics analysis and literature review. We summarize the core clinical features and standardized management of this disease to provide a practical reference for pediatric CN diagnosis and care. This study was approved by the Ethics Committee of Guangyuan Central Hospital, and written informed consent was obtained from the patient’s legal guardian.

## 2 Case presentation

### 2.1 General information

A 7−year−old girl was admitted to the First Pediatric Department, Women’s and Children’s Health Branch of Guangyuan Central Hospital on June 5, 2025, with a 4−day history of fever and 1−day history of perianal swelling and pain. She was the second child of healthy non−consanguineous parents, delivered at term by cesarean section with a birth weight of 2.75 kg and no birth asphyxia. Her older brother died in a traffic accident. There was no family history of recurrent fever or hematologic disease.

## 3 Clinical history

### 3.1 Present illness

Four days before admission, she developed unexplained fever (peak 39.0 °C) with neck pain and oral discomfort, without cough, wheezing, rhinorrhea, vomiting, diarrhea, convulsions, or rash. Oral cephalosporin treatment was ineffective. One day before admission, perianal swelling and left inguinal pain developed and did not improve with continued antibiotics. A complete blood count (CBC) at Guangyuan Traditional Chinese Medicine Hospital showed ANC 0.04×10^9^/L, prompting transfer to our hospital for further evaluation.

### 3.2 Past medical history

She had occasional unexplained fever since infancy, with recurrent febrile episodes starting in September 2022. Febrile periods consistently showed neutropenia (lowest ANC 0.17×10^9^/L on March 16, 2024) and monocytosis, with granulocyte counts normalizing between episodes. Initially, fever occurred non−periodically every 2–3 months (peak 38.5–39.0 °C, duration 3–5 days) and resolved with oral antibiotics and antipyretics, but no etiology was identified. Since March 2024, episodes became regularly periodic (initially monthly, then every 2 weeks after September 2024) with worsening symptoms including recurrent oral ulcers and gingival swelling. No systematic evaluation was performed before this admission, and the current episode was complicated by new−onset perianal swelling and pain.

### 3.3 Physical examination

Vital signs: temperature 36.7 °C, heart rate 95 beats/min, respiratory rate 18 breaths/min, weight 19 kg, height 120 cm. Physical development and nutritional status were normal; she was alert and in fair general condition, with pink complexion, no dehydration, good skin turgor, and no rash.

Bilateral cervical lymphadenopathy was present (largest node 1.5 cm × 2 cm, moderate consistency, limited mobility, warm and tender, no fluctuation). A tender left inguinal lymph node (1.0 cm × 0.8 cm) was also palpable. Conjunctivae were not injected; lips were pink, tongue pale red with mild papillary swelling. A 5−mm superficial ulcer on the right upper gingiva was noted, with gingival atrophy, erythema, swelling, and easy bleeding. Pharynx was congested; tonsils were not enlarged and showed no purulent exudate.

Neck was supple. Bilateral breath sounds were slightly coarse without rales. Heart sounds were strong and regular with no murmur. Abdomen was soft, non−tender, with no hepatosplenomegaly. A 1−cm tender, firm, fixed mass with overlying erythema and warmth was palpable at the 3−o’clock perianal position, without fluctuation. Extremity joint movement, skin perfusion, nailfold circulation, and peripheral pulses were normal. Neurologic examination was unremarkable.

### 3.4 Laboratory examinations

All hematologic, biochemical, and ancillary test results are integrated into a single transposed table with pediatric reference ranges for direct comparison (**Table 1**).

**Table 1:** Comprehensive laboratory findings.

Parameter	Unit	Pediatric reference range	2025-06-05Guangyuan TCM Hospital	2025-06-07Guangyuan Central Hospital
White blood cell (WBC)	×10^9^/L	4.0–10.0	5.17	4.34
Absolute neutrophil count (ANC)	×10^9^/L	1.5–7.0	0.04 ↓	0.55 ↓
Neutrophil ratio	%	50.0–70.0	0.7 ↓	12.6 ↓
Monocyte count	×10^9^/L	0.12–1.0	2.31 ↑	1.67 ↑
Monocyte ratio	%	3.0–12.0	44.7 ↑	38.4 ↑
Hemoglobin (Hb)	g/L	110–140	115	–
Platelet (PLT)	×10^9^/L	100–300	209	–
High-sensitivity C-reactive protein (hs-CRP)	mg/L	0–5	34.74 ↑	40.71 ↑
Serum 25-hydroxyvitamin D	ng/mL	≥20.0	19.52 ↓	–
Liver/renal function	–	Normal	Normal	Normal
Electrolytes	–	Normal	Normal	Normal
Cardiac enzymes	–	Normal	Normal	Normal
Coagulation profile	–	Normal	Normal	Normal
Blood culture	–	Negative	Negative	–
Throat swab culture	–	Negative	Negative	–
Urinalysis	–	Normal	Normal	Normal
Stool routine	–	Normal	Normal	Normal
IgG, IgA, CD4^+^/CD8^+^	–	Normal	Normal	Normal
Bone marrow smear	–	Normal hematopoiesis	Active hematopoiesis; granulocytic toxic changes	–

↓ below reference range; ↑ above reference range; – not tested.

### 3.5 Genetic Testing

Whole−exome sequencing (WES) performed at West China Second University Hospital, Sichuan University on June 27, 2025, detected a heterozygous missense variant in *ELANE* (NM_001972.4: c.416C>T, p.Pro139Leu). Parental testing at this locus was negative, confirming a *de novo* origin. According to ACMG guidelines, this variant was classified as pathogenic (7, 19–21).

**Figure 1:**
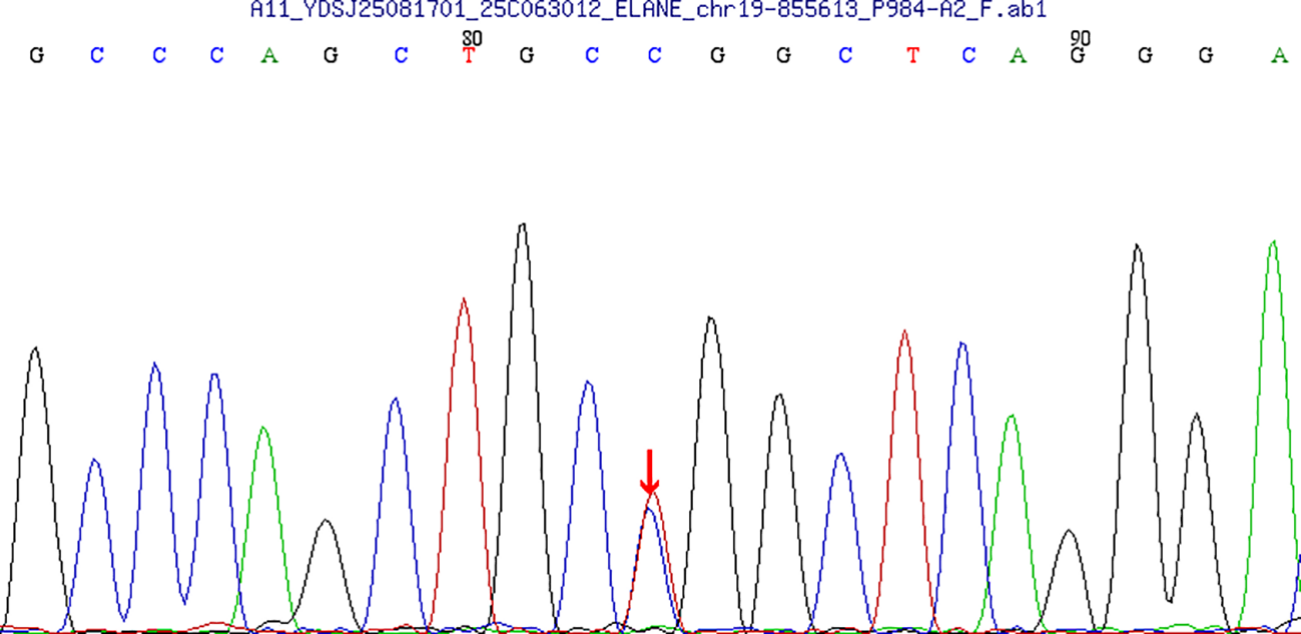
Sanger sequencing of the *ELANE* geneArrow indicates the heterozygous c.416C>T variant in the patient, resulting in p.Pro139Leu substitution.

Sanger sequencing validation at Beijing Mageno Medical Laboratory on August 19, 2025, confirmed the heterozygous *ELANE* c.416C>T variant with clear sequencing peaks (**Figure 1**).

Subject: c.416C>T heterozygous mutation.

### 3.6 Dynamic peripheral blood count monitoring

**Figure 2:**
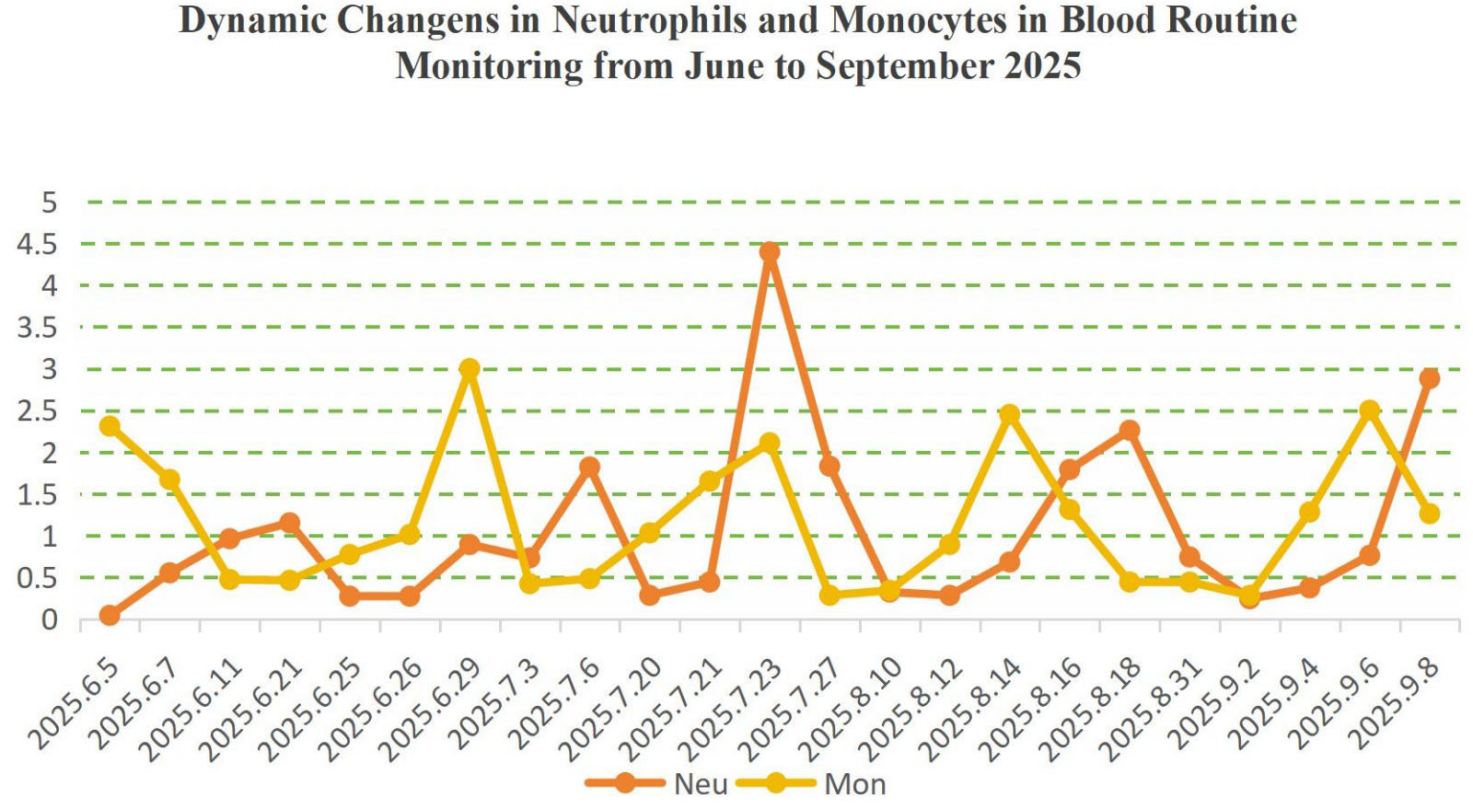
Dynamic changes in peripheral blood neutrophil and monocyte counts (June–September 2025)Neutrophils showed regular 21−day cycles, repeatedly dropping below 0.5×10^9^/L at nadir. Monocyte counts rose reciprocally during neutropenic nadirs, consistent with periodic fever—a classic laboratory signature of CN.

Serial CBC monitoring from June to September 2025 confirmed regular 21−day ANC cycles. ANC repeatedly fell below 0.5×10^9^/L at nadir, with reciprocal monocyte elevation synchronous with febrile episodes (**Figure 2**). ANC <0.5×10^9^/L represents a high infection risk threshold in children; the normal pediatric ANC range is 1.5–7.0×10^9^/L.

## 4 Treatment and follow−up

### 4.1 Initial treatment

Empirical anti−infective therapy with cefazolin sodium was started on admission. Adjunctive care included chlorhexidine mouth rinse, mupirocin ointment for perianal lesions, antipyretics, and fluid replacement. Symptoms gradually improved.

After genetic confirmation, the patient was referred to the Hematology Department of West China Second University Hospital. Comprehensive evaluation recommended HSCT, and she is currently awaiting a matched donor. This strategy was chosen due to long−term risks of persistent neutropenia (recurrent severe infection, low−risk myelodysplastic syndrome [MDS] transformation) and the curative potential of HSCT for monogenic hematologic disorders. For acute episodes, subcutaneous rhG−CSF is administered at 5 μg/(kg·d) daily until ANC exceeds 0.5×10^9^/L (typical 3–5 days per course), combined with intensified anti−infective and supportive care.

## 5 Follow−up outcomes

As of September 2025, the patient’s cycle length remains stable at approximately 21 days. Infections are rapidly controlled with rhG−CSF; no severe complications (sepsis, necrotizing enterocolitis) have occurred. Growth and development are within normal limits, and regular long−term follow−up is ongoing.

## 6 Discussion

CN is a rare congenital hematopoietic disorder (incidence ~1 per million) first reported in 1910 (22, 23). Its core pathogenesis is impaired granulocyte maturation caused by *ELANE* mutations (19p13.3) (4, 24, 25). CN is mostly autosomal dominant, but *de novo* mutations also occur, as in this patient (7, 21). Identification of the pathogenic heterozygous *ELANE* c.416C>T variant provided definitive molecular confirmation.

Bioinformatics analysis showed the Pro139 residue of neutrophil elastase is highly conserved across species (26, 27). The Pro139Leu substitution alters side−chain structure, likely disrupting protein conformation and enzymatic activity, thus interfering with myeloid differentiation and granulocyte maturation—consistent with the known pathogenic mechanism of *ELANE* mutations (8). The *ELANE* c.416C>T (p.Pro139Leu) variant has been reported in both SCN and CN (20). Published cases include a 2−year−old girl with recurrent oral ulcers and infection (19), and a familial case with maternal perirectal abscess, anal fistula, and gingivitis onset at 28 years, with similar childhood features in her daughter (7). Infectious manifestations (oral mucosa, skin, perianal region) closely match our patient.

Notably, prior cases with this variant showed poor rhG−CSF response, requiring dose escalation to 30–83 μg/kg/d. In contrast, our patient achieved effective control with a low dose of 5 μg/kg/d (28). This marked difference indicates substantial genotype–phenotype heterogeneity, possibly related to individual genetic background and modifier genes (7, 21).

The classic CN feature is 21−day ANC cycling (range 14–35 days (25, 29); rarely up to 90 days). ANC typically falls below 0.2×10^9^/L for 3–6 days before spontaneous recovery, with symptom−free intervals (30). Infections predominantly involve mucosal and cutaneous sites: fever, oral ulcers, gingivitis, lymphadenopathy, and skin lesions (23, 31, 32). Perianal cellulitis is common in children; severe cases may progress to life−threatening sepsis, necrotizing enterocolitis, or peritonitis—the main causes of mortality (33, 34). CN usually presents in the first year of life, but childhood and adult onset are well documented (33, 35). Our patient developed fever and neutropenia at 4 years 7 months, with typical cyclic symptoms (fever, pharyngalgia, lymphadenopathy, gingivitis, stomatitis, neutropenia) emerging from March 2024, complicated by perianal cellulitis. Episodes recurred every 2–4 weeks with complete remission between cycles—fully consistent with CN. Diagnostic delay resulted from low disease awareness and lack of serial granulocyte monitoring.

*ELANE* is the major causative gene for CN, with a pathogenic variant detection rate of 80%–100% in clinically typical cases (20, 36). Genetic testing is essential for confirmatory diagnosis and variant identification (30, 37). CN is autosomal dominant; *de novo* mutations are reported but with unknown exact frequency (37, 38). Pathogenic variant carriers have a 50% offspring inheritance risk (28). Our patient had no relevant family history, and WES confirmed a *de novo* heterozygous *ELANE* c.416C>T (p.Pro139Leu) mutation. Other neutropenia−associated genes include CSF3R and HAX1, but *ELANE* is predominant in CN (3, 13, 39). The neutropenic nadir is a high−risk infection window, with rapid local inflammation progression and potential life−threatening sepsis.

Definitive CN diagnosis requires three criteria (40–43): (1) typical periodic fever and mucosal/cutaneous infection; (2) serial CBC monitoring (2–3 times weekly for 6–8 weeks) confirming cyclic ANC fluctuation; (3) detection of a pathogenic ELANE variant. Diagnostic challenges include rarity, low clinical awareness, nonspecific early symptoms, and need for prolonged monitoring, leading to misdiagnosis as common infection. CN should be strongly suspected in children with recurrent periodic infection and fluctuating neutropenia, with early genetic testing for confirmation.

Key differential diagnoses (44–47):

SCN: Persistently low ANC without cycling; ELANE−related cases show more severe infection and higher deep abscess risk.

Autoimmune neutropenia: Often with thrombocytopenia; anti−neutrophil antibodies are diagnostic.

Periodic fever syndromes: Sustained inflammatory elevation without cyclic neutropenia; confirmed by MEFV, TNFRSF1A, etc.

## 7 Treatment and management strategies

According to the 2022 Chinese Expert Consensus on the Diagnosis and Treatment of Neutropenia, CN management focuses on infection control and ANC elevation (48):

rhG−CSF is first−line for patients with recurrent infection or ANC <0.5×10^9^/L. The minimal effective dose should maintain ANC >1.0×10^9^/L to reduce infection frequency and severity (49, 50).

Prompt broad−spectrum antibiotics for acute infection, plus enhanced oral, skin, and perianal care to prevent progression.

Long−term follow−up: Bone marrow morphology every 3–6 months to screen for MDS transformation (incidence ~1%–3%).

HSCT is curative for rhG−CSF−refractory or severe CN.

Our patient responded well to low−dose rhG−CSF; HSCT is planned to mitigate long−term risks (recurrent severe infection, MDS transformation) and achieve cure. This is a proactive long−term strategy, not an urgent indication (48).

The patient was followed for 3 months (through September 2025) with stable status. Long−term care must prioritize MDS surveillance: quarterly bone marrow morphology and ELANE/CSF3R testing, plus semi−annual liver/renal and hematopoietic function assessment. Adverse effects of long−term rhG−CSF (bone pain, splenomegaly) require close monitoring; dose dependency or reduced efficacy should prompt timely regimen adjustment (20, 51, 52, 53).

This *de novo* ELANE c.416C>T (p.Pro139Leu) variant has been reported in four prior cases, all linked to severe neutropenia and confirmed pathogenicity. This case expands the clinical phenotypic spectrum of ELANE-related CN, especially the favorable low−dose rhG−CSF response in a late−childhood−onset patient (4 years 7 months). It also underscores that CN should be included in the differential diagnosis of children with recurrent infection and fluctuating neutropenia regardless of onset age. Timely integrated clinical and genetic evaluation is critical for accurate diagnosis and standardized management.

## Data Availability

The original contributions presented in the study are included in the article/supplementary material. Further inquiries can be directed to the corresponding author.

## Glossary

ANC: Absolute Neutrophil Count
CN: Cyclic Neutropenia
SCN: Severe Congenital Neutropenia
rhG-CSF: Recombinant Human Granulocyte Colony-Stimulating Factor
HSCT: Hematopoietic Stem Cell Transplantation
MDS: Myelodysplastic Syndrome
WBC: White Blood Cell
RBC: Red Blood Cell
Hb: Hemoglobin
PLT: Platelets
hs-CRP: High-sensitivity C-reactive Protein
SAA: Serum Amyloid A
PCT: Procalcitonin.
